# Analysis of CT morphologic features and attenuation for differentiating among transient lesions, atypical adenomatous hyperplasia, adenocarcinoma *in situ*, minimally invasive and invasive adenocarcinoma presenting as pure ground-glass nodules

**DOI:** 10.1038/s41598-019-50989-1

**Published:** 2019-10-10

**Authors:** Lin Qi, Ke Xue, Cheng Li, Wenjie He, Dingbiao Mao, Li Xiao, Yanqing Hua, Ming Li

**Affiliations:** 1 Department of Radiology, Huadong Hospital affiliated to Fudan University, Shanghai, China; 2 Department of Pathology, Huadong Hospital affiliated to Fudan University, Shanghai, China; 3 Department of Plastic and Reconstructive Surgery, Shanghai 9th People’s Hospital, Shanghai Jiao Tong University School of Medicine, Shanghai, China

**Keywords:** Cancer imaging, Lung cancer

## Abstract

Thin-section computed tomography (TSCT) imaging biomarkers are uncertain to distinguish progressive adenocarcinoma from benign lesions in pGGNs. The purpose of this study was to evaluate the usefulness of TSCT characteristics for differentiating among transient (TRA) lesions, atypical adenomatous hyperplasia (AAH), adenocarcinoma *in situ* (AIS), minimally invasive adenocarcinoma (MIA) and invasive adenocarcinoma (IAC) presenting as pure ground-glass nodules (pGGNs). Between January 2016 and January 2018, 255 pGGNs, including 64 TRA, 22 AAH, 37 AIS, 108 MIA and 24 IAC cases, were reviewed on TSCT images. Differences in TSCT characteristics were compared among these five subtypes of pGGNs. Logistic analysis was performed to identify significant factors for predicting MIA and IAC. Progressive pGGNs were more likely to be round or oval in shape, with clear margins, air bronchograms, vascular and pleural changes, creep growth, and bubble-like lucency than were non-progressive pGGNs. The optimal cut-off values of the maximum diameter for differentiating non-progressive from progressive pGGNs and IAC from non-IAC were 6.5 mm and 11.5 mm, respectively. For the prediction of IAC vs. non-IAC and non-progressive vs. progressive adenocarcinoma, the areas under the receiver operating characteristics curves were 0.865 and 0.783 for maximum diameter and 0.784 and 0.722 for maximum CT attenuation, respectively. The optimal cut-off values of maximum CT attenuation were −532 HU and −574 HU for differentiating non-progressive from progressive pGGNs and IAC from non-IAC, respectively. Maximum diameter, maximum attenuation and morphological characteristics could help distinguish TRA lesions from MIA and IAC but not from AAH. So, CT morphologic characteristics, diameter and attenuation parameters are useful for differentiating among pGGNs of different subtypes.

## Introduction

The Fleischner Society Guidelines issued in 2013 proposed substantive changes in the follow-up and management of sub-solid nodules. In the revised guidelines, pGGNs equal to or less than 5 mm in diameter do not need routine follow-up. Five-year follow-up scanning is recommended for pGGNs with diameters equal or greater than 6 mm^1^. The 2015 World Health Organization Classification of Lung Tumours suggests that on TSCT, pGGNs typically indicate the presence of preinvasive lesions, while invasive adenocarcinoma usually manifests as part-solid GGNs and solid nodules^2^. If completely resected, the 5-year survival rates of patients with AIS and MIA can reach 100% and near 100%, respectively, whereas the 5-year survival rate of patient with IAC is 74.6%^3^.

With the widespread use of clinical TSCT screening, a growing number of pGGNs have been detected, and MIA and IAC are usually pathologically found in pGGNs^4–6^. Because of the absence of a measurable solid component, we have to search for other imaging biomarkers to distinguish progressive adenocarcinoma from benign lesions. Therefore, the aim of the present study was to retrospectively investigate morphologic features of different pathological subtypes presenting as pGGNs on TSCT.

## Patients and Methods

The Institutional Review Board of Huadong hospital affiliated Fudan University approved our study with a waiver of the requirement for patient informed consent (approval no. 20170721). All methods were performed in accordance with the relevant guidelines and regulations.

### Patient selection

**Figure 1:**
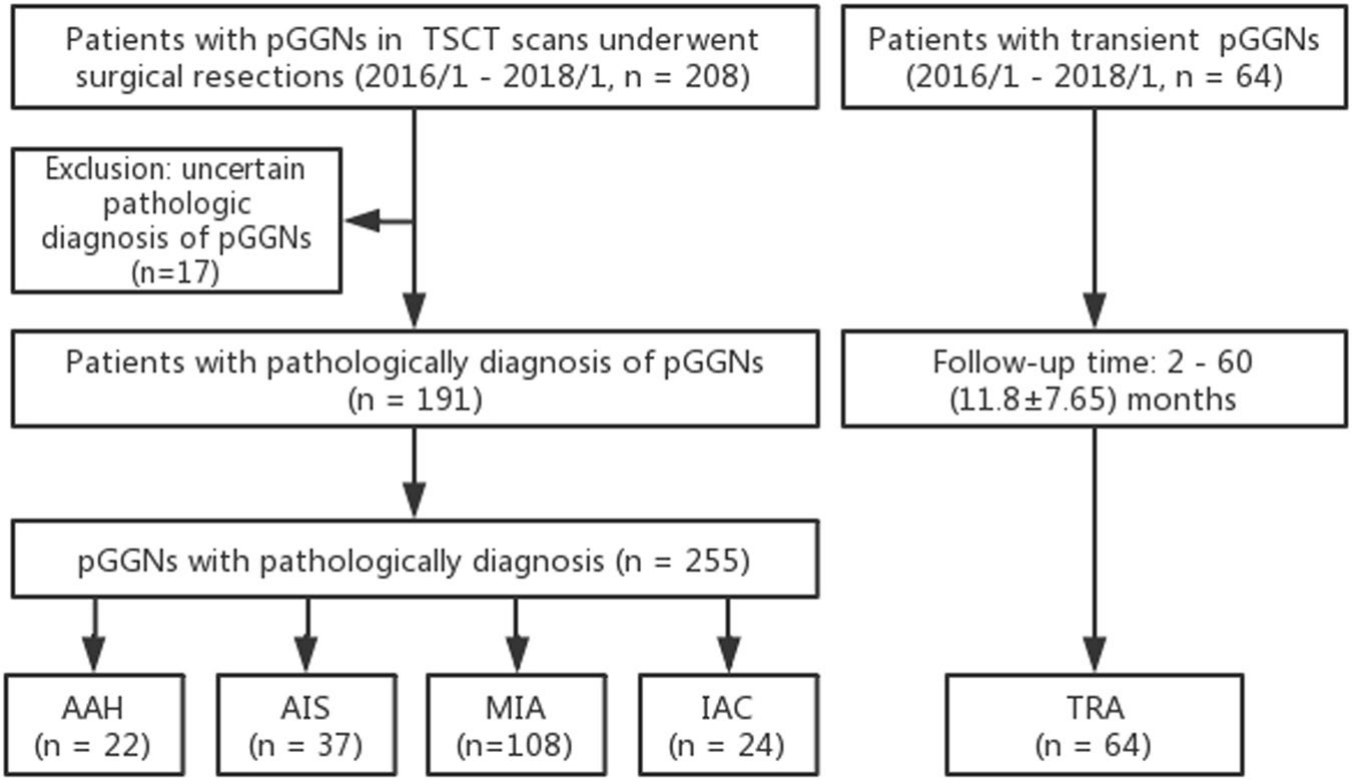
Flowchart of research participants examined by TSCT and pathology. TRA: transient pGGNs; AAH: atypical adenomatous hyperplasia; AIS: adenocarcinoma *in situ*, MIA: minimally invasive adenocarcinoma; IAC: invasive adenocarcinoma. pGGNs: pure ground glass nodules.

We retrospectively reviewed TSCT (≤1.25 mm) images of 208 patients who had pGGNs in preoperative CT scans and subsequently underwent surgical resections at our hospital between January 2016 and January 2018. All of the patients had definite diagnoses based on the pathological examination of surgical specimens. The mean interval between the last CT examination and surgical resection was 16 days. Among the 208 patients, 17 (8.2%) were excluded because of uncertain pathologic diagnosis. Because those pGGNs were found in the same lobe of the lung as the dominant nodules and failed to be pathologically examined after lobectomy. Consequently, a total of 191 surgically resected pGGNs were included in our study and 255 pGGNs were included in our research. Among the 191 patients, 66 (34.6%) underwent lobectomy, and 125 (65.4%) underwent segmentectomy. In addition, we gathered 64 transient (TRA) solitary pGGNs with diameters less than 30 mm in 64 patients who did not display any symptoms of fever, cough and expectoration. These pGGNs were all found by chance during periodic health check-ups or on CT examination of unrelated pulmonary or extra-pulmonary diseases. They were difficult to differentiate from persistent pGGNs, and some of them even mimicked MIA or IAC. After 2–60 months (11.8 ± 7.65) of follow-up, all of these nodules disappeared and were included in our study as the TRA group (Fig. 1).

The inclusion criteria for our study were as follows: (1) lesions manifesting as pGGNs on TSCT, (2) the maximum diameter of pGGNs ≤30 mm, and 3) surgically resected pGGNs with a definite pathological diagnosis. Persistent pGGNs found on TSCT, which were being follow-up and had not been confirmed pathologically, were not included in our study. During the period from January 2016 to January 2018, pathology revealed 4 benign pGGNs, including 2 fibrous proliferations, 1 hamartoma, and 1 papillary adenoma. These nodules were not included in our analysis because the number of cases was too small.

### CT protocols and image analysis

CT scans were obtained with 64-slice Discovery CT750 HD (GE Healthcare) and Siemens SOMATOM Definition Flash scanners (Munich, Germany) using similar protocols as follows: tube voltage, 120 kVp; body mass index (BMI) – dependent tube current, 220 mAs for BMI ≤25 kg/m^2^ and 330 mAs for BMI >25 kg/m^2^; collimator, 64 × 0.625 mm for GE scanner and 64 × 0.6 mm for Siemens scanner. The images were reconstructed into 0.625 mm and 1 mm slice thickness for GE and Siemens images, respectively. Two observers with 12 and 14 years of experience in chest CT interpretation analysed the morphologic manifestations of all pGGNs on axial and reconstructive images based on the pre-defined criteria and recorded them in an Excel spreadsheet. Morphology included shape (round, oval or irregular), margin (clear, unclear, or fuzzy), vascular change (normal, convergent or dilated), pleural change (normal or involved), sign of creep growth, bubble-like lucency, and air bronchogram. Creep growth was defined as a pGGN next to larger pulmonary vessels, without adventitia invasion. Bubble-like lucency was defined as air attenuation within the pGGNs. Air bronchograms reflected the dilation of small bronchi passing though the pGGNs. And these two observers also measured CT attenuation values on the axial lung window images before surgery using region-of-interest (ROI) cursors, which were drown manually and covered two-thirds of the largest area in the pGGN away from air space.

Five promising quantitative parameters were evaluated and calculated on TSCT: size (maximum diameter on axial images), maximum CT value, lung parenchyma CT value, difference CT value, and relative attenuation. The maximum tumour size was represented by the maximum tumour diameter measured on the same lung window setting (width, 1500 HU; level, −500 HU). CT attenuation was manually measured five times in the highest density area seen within the nodule by naked eye while avoiding small blood vessels. We took the crest value as the maximum CT attenuation of the nodule. The lung parenchyma CT value was measured in the normal area of lung tissues 10 mm from the edge of the nodule, avoiding blood vessels and bronchi. The difference CT value was the maximum CT value minus the lung parenchyma CT value. The relative attenuation was calculated by dividing the lung parenchyma CT value by the maximum CT attenuation.

### Selection of treatment and pathological analysis

All pathological specimens were obtained by video-assisted thoracoscopy or thoracotomy. Specimens were fixed in 10% formalin and embedded in paraffin. Several sections were taken from the middle of the lesions and then stained with haematoxylin-eosin. Two pathologists with 7 and 12 years of experience in lung pathology assessed the specimen independently, blinded to radiological features, and decisions on pathological diagnosis were reached by consensus. Adenocarcinomas were histologically re-evaluated on the basis of the multidisciplinary classification proposed by the International Association for the Study of Lung Cancer/American Thoracic Society/European Respiratory Society in 2011^7^.

### Statistical analysis

Statistical analysis was carried out by SPSS 22.0 software and GraphPad Prism. Intraclass correlation coefficients (ICCs) and 95% Bland-Altman limits of agreement were used to assess inter-observer variability for the measurements of pGGNs on TSCT. Correlation was graded as follows: ICC 0–0.20, poor; ICC 0.21–0.40, fair; ICC 0.41–0.60, moderate; ICC 0.61–0.80, good; and ICC 0.81–1.00, excellent. Values were described with either mean ± standard deviation (SD) or median with interquartile range (IQR) after testing the normality of variables using Shapiro-Wilk test. Then those data were compared by using Pearson chi-squared test and Kruskal-Walls test for categorical variables (gender, location, distribution, shape, margin, air bronchogram, vessel change, pleural change, creep growing sign, bubble-like lucency) and continuous variables (age, size, volume, maximum CT value, lung parenchyma CT value, relative CT value, difference CT value), respectively. We used Dunnett t-test to treat ICA or TRA as a control group, and then compared all other groups against it. We used logistic analysis for the univariate analysis and multivariate regression analysis to identify the independent predictors of progressive adenocarcinomas, using IAC and progressive adenocarcinoma (MIA plus IAC) as outcome variables and lesion characteristics as potential predictors, respectively. For the progressive/ none-progressive groups, Mann-Whitney U Test was performed to compare the differences of continuous variables, and Pearson chi-square test for categorical variables. Receiver operating characteristic (ROC) analysis was used to determine a cutoff value of size and attenuation parameters. A *P* value of less than 0.05 was considered significant.

## Results

### Descriptive statistics and TSCT findings

**Figure 2:**
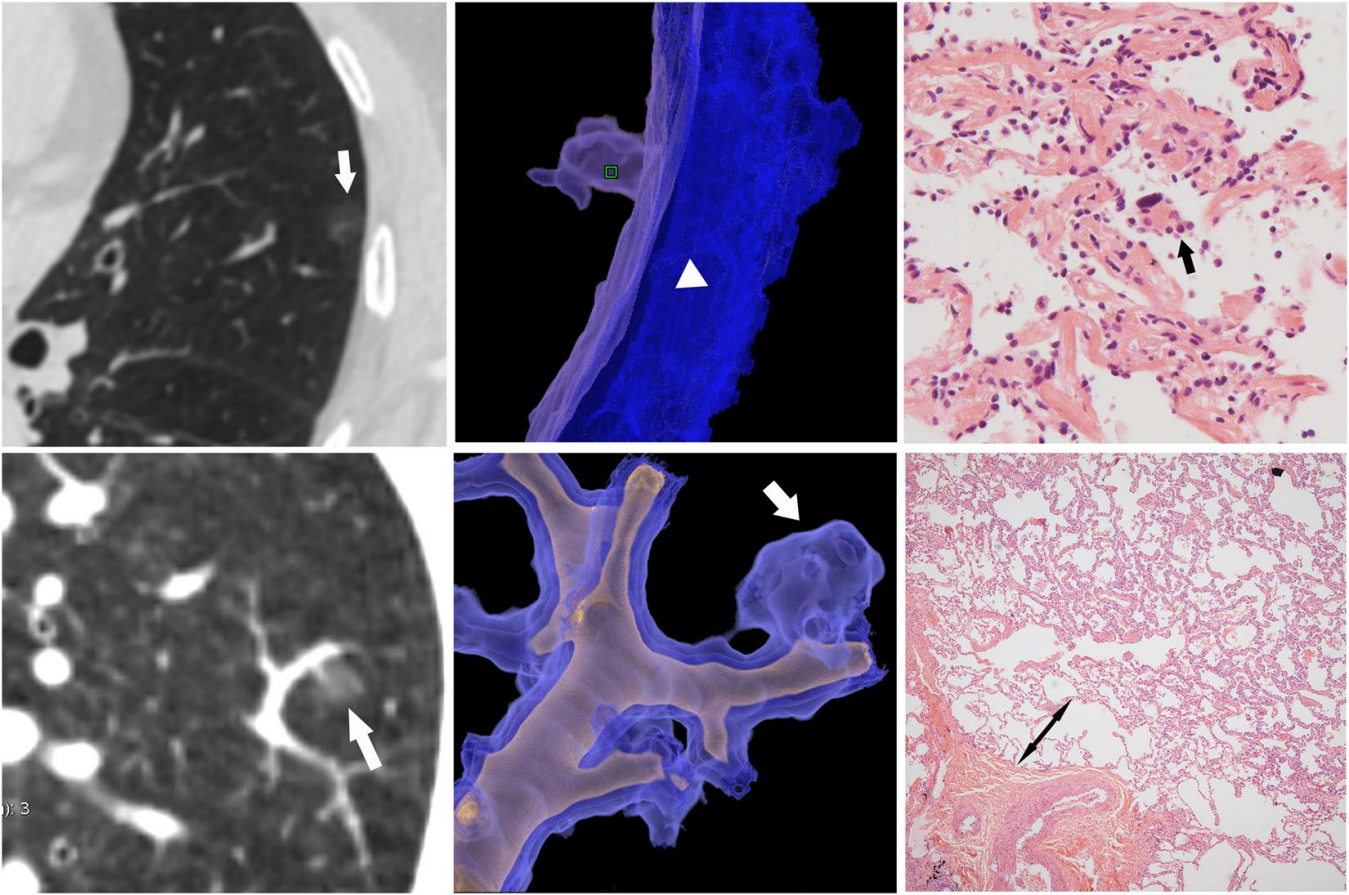
(**a**∼**c**) AAH in a 58-year-old woman. **(a)** Axial CT image shows the nodule is located in the upper lobe of the left lung (white arrow), with an maximum diameter of 6 mm. The maximum CT attenuation is low (−708HU) which suggested benign character. (**b**) Reconstructive CT image demonstrates the pGGN is close to pleura without pleura invasion. **(c**) High-power photomicrograph (Hematoxylin-Eosin, 100×) shows the proliferation and slight atypicality of epithelial cells (black arrow) along the alveolar space, and the cells are closely arranged with each other without any overlap and extrusion. (**d**∼**f**) AIS in a 49-year-old man. **(d**) Reconstructive CT image shows the pGGN (white arrow) close to a pulmonary vessel but there is a small gap with the adventitia. The maximum diameter is 6 mm, and the maximum CT value is −619HU. (**e**) Volume rendering image shows the vessel walking naturally with dilated and wrapped around. (**f**) Photomicrograph of a histologic specimen (Hematoxylin-Erosin, 40×) shows there is a space of normal lung parenchymal structure between the nodule edge and the vessel adventitia. (double-sided arrow).

**Figure 3:**
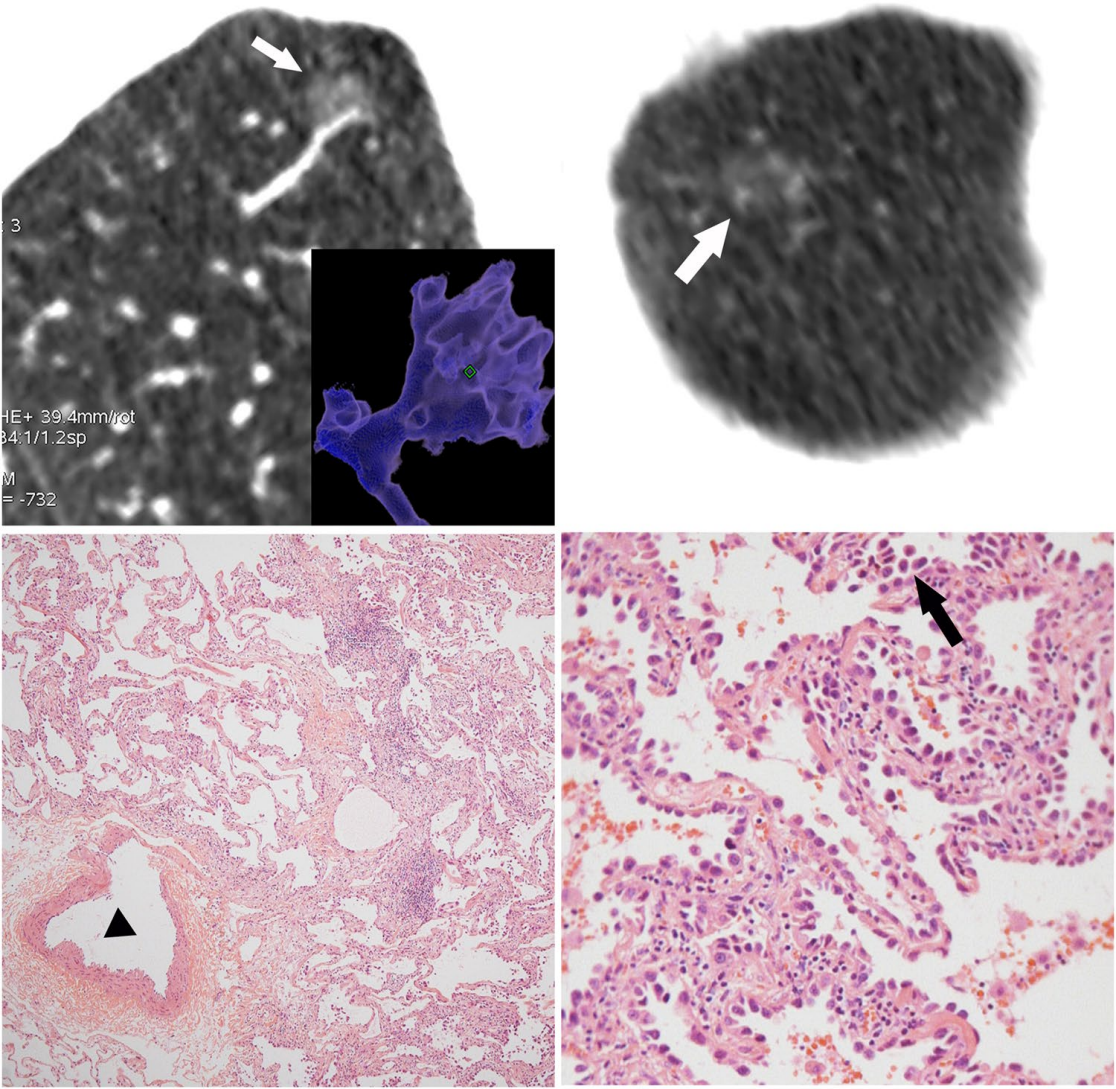
MIA in a 56-year-old woman. **(a)** Reconstructive CT image shows the typical creep growing sign (white arrow). The nodule is in close contact with the adventitia of pulmonary blood vessel without any space. The maximum diameter is 11 mm, and the maximum CT attenuation is relatively higher (−573). The creep growing sign and higher maximum CT value predict malignancy. (**b)** Axial CT image shows the cross section of the pulmonary vessel with slight dialation. (**c)** Photomicrograph of a histologic specimen (Hematoxylin-Erosin, 40×) shows the cross section of the same pulmonary vessel (▲). The nodule-vessel interface is clear without infiltration. (**d)** High-power photomicrograph (Hematoxylin-Eosin, 200×) shows the lepidic and stacking growth pattern (black arrow) of tumor cells invading into the fibrous stroma.

**Figure 4:**
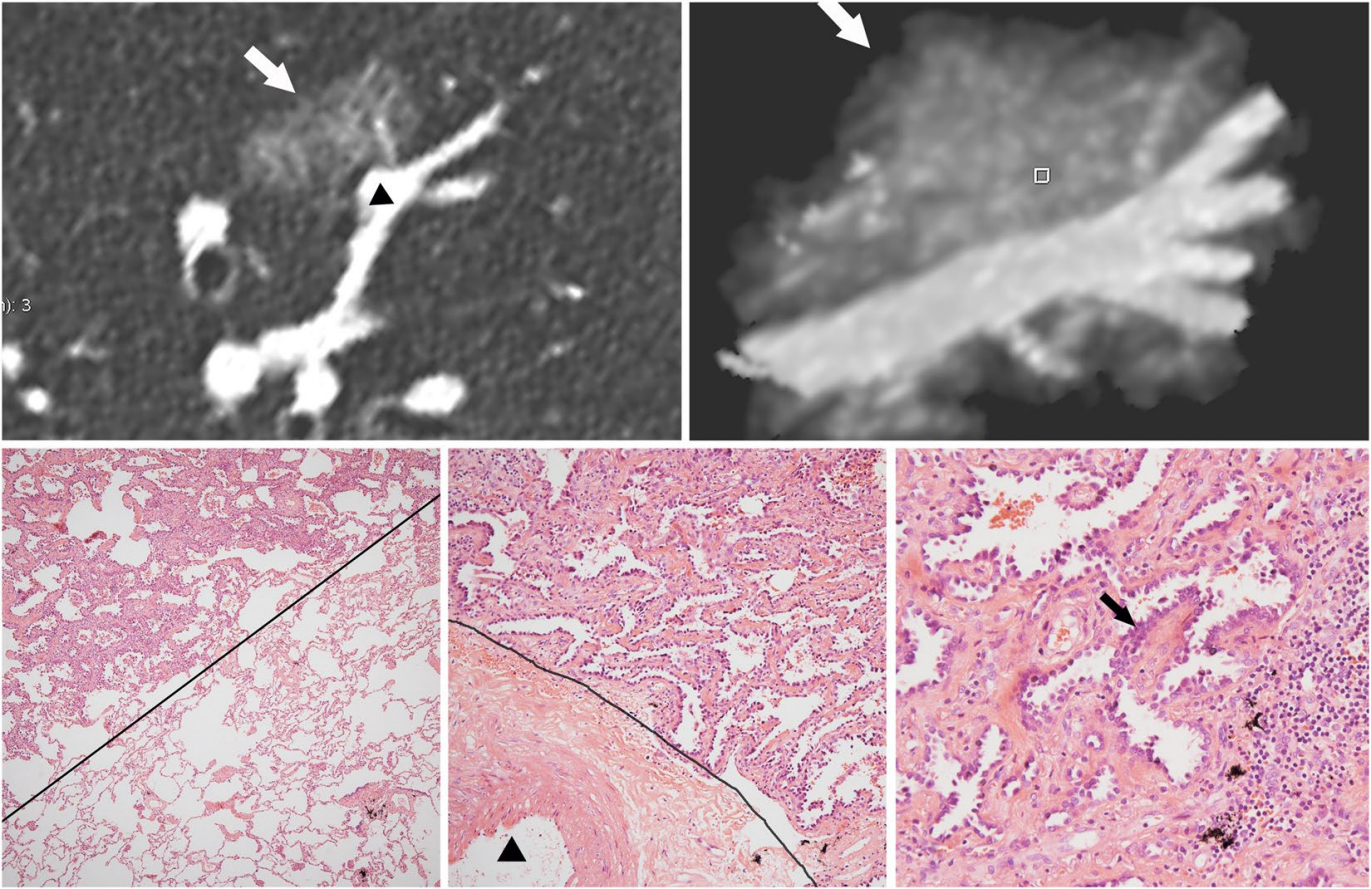
IAC in a 63-year-old man. **(a)** Reconstructive CT image showing typical creep growth (white arrow) and clear edge of the pGGN. The maximum diameter is 17 mm, and the maximum CT attenuation is relatively high (−552 HU). (**b)** Maximum intensity projection image showing a clear nodule-vessel interface. (**c)** Low-magnification image of a histologic specimen (haematoxylin-eosin, 40×) showing a clear boundary between the nodule edge and normal parenchymal structures (dotted black line). (**d)** High-magnification image (haematoxylin-eosin, 100×) showing a clear boundary between the nodule edge and the vessel adventitia (dotted black line). (**e)** High-magnification image (haematoxylin-eosin, 200×) showing crowded and overlapping tumour cells growing in clusters into the lumen (black line) with interstitial fibroblast involvement (▲).

The descriptive statistics of the features in the five groups are summarized in Table 1. A total of 255 pGGNs from 191 patients were evaluated including 64 TRA, 22 AAH, 37 AIS, 108 MIA and 24 IAC cases (Figs 2–4). None of the patients had lymph node metastasis based on histopathological examination of the surgical specimens. Among the five groups, age, sex, bubble-like lucency, difference CT value and relative CT value displayed significant differences. The maximum diameter, shape, margin, air bronchogram, vascular change, creep growth, and maximum diameter showed significantly higher differences than the above parameters. No significant differences were detected regarding lobe location or lung parenchyma CT value. The maximum diameter and maximum CT attenuation exhibited an increasing trend according to the pathologic disease severity from AAH to IAC. The relative CT value and difference CT value were no more effective than the maximum diameter in indicating the risk of pGGNs.

**Table 1:** Radiologic characteristics of study population in TRA∼IAC groups.

	Total	TRAs	AAHs	AISs	MIAs	IACs	H	p
pGGNs n [%]	255	64 [25.1]	22 [8.6]	37 [14.5]	108 [42.4]	24 [9.4]	\	\
Age (years)	55 (43–62)	51 ± 13	57 ± 9	52 ± 13	55 (41–63)	61 ± 10	13.47	0.009^**^
Gender male [%]	80 [31.4]	27 [42.2]	3 [13.6]	10 [27]	27 [25]	13 [54.2]	14.84	0.005^**^
Lobe location							14.12	0.590
RUL	92	17	5	16	43	11		
RML	28	8	3	3	12	2		
RLL	44	12	4	8	18	2		
LUL	60	15	9	6	24	6		
LLL	31	12	1	4	11	3		
Location							18.4	0.018^*^
Inner	34 [13.3]	10	3	4	13	4		
Middle	108 [42.4]	38	5	13	40	12		
Peripheral	113 [44.3]	16	14	20	55	8		
Nodule size (mm)	7 (5–9)	5 (4–6)	5 (4–6.5)	7 (5.5–8)	13 (11.3–17)	14 ± 5.7	90.77	0.000^****^
Shape							28.54	0.000^****^
Round or oval	88	9	4	13	46	18		
Irregular	167	55	18	24	62	8		
Margin							37.23	0.000^****^
Clear	161	22	17	24	83	15		
Unclear	66	29	5	11	16	5		
Fuzzy	28	0	0	2	9	4		
Air bronchogram	31	2	1	2	13	13	47.33	0.000^****^
Vessel change							80.43	0.000^****^
Normal	149	59	18	29	39	4		
Gather/Dilated	106	5	4	8	69	20		
Pleural change	27	2	0	2	15	8	21.78	0.000^****^
Creep growing sign	63	2	1	8	43	9	36.38	0.000^****^
Bubble-like lucency	21	0	1	0	12	8	30.65	0.001***
Maximum CT value	−585 (−668–475)	−655 (−736–588)	−656 (−710–572)	−587 ± 103	−519 (−639–439)	−457 ± 105	64.77	0.000^****^
Lung parenchyma CT value	−881 (−899–857)	−874 ± 35	−881 (−899–856)	−877 ± 28	−883 (−901–860)	−877 ± 30	1.036	0.904
Difference CT value	290 (201–404)	209 (144–295)	218 (173–283)	263 (230–332)	359 (245–441)	402 (331–543)	66.7	0.000**^**^
Relative CT value	1.49 (1.30–1.83)	1.32 (1.20–1.48)	1.33 (1.25–1.50)	1.42 (1.33–1.62)	1.69 (1.40–2.00)	1.85 (1.69–2.95)	67.55	0.000****

RUL, right upper lobe; RML, right middle lobe; RLL, right lower lobe; LUL, left upper lobe; LLL, left lower lobe. Significant level marks: p < 0.05 *p < 0.01 **p < 0.001 ***p < 0.0001 ****.

### Inter-observer agreement of TSCT measurements

**Figure 5:**
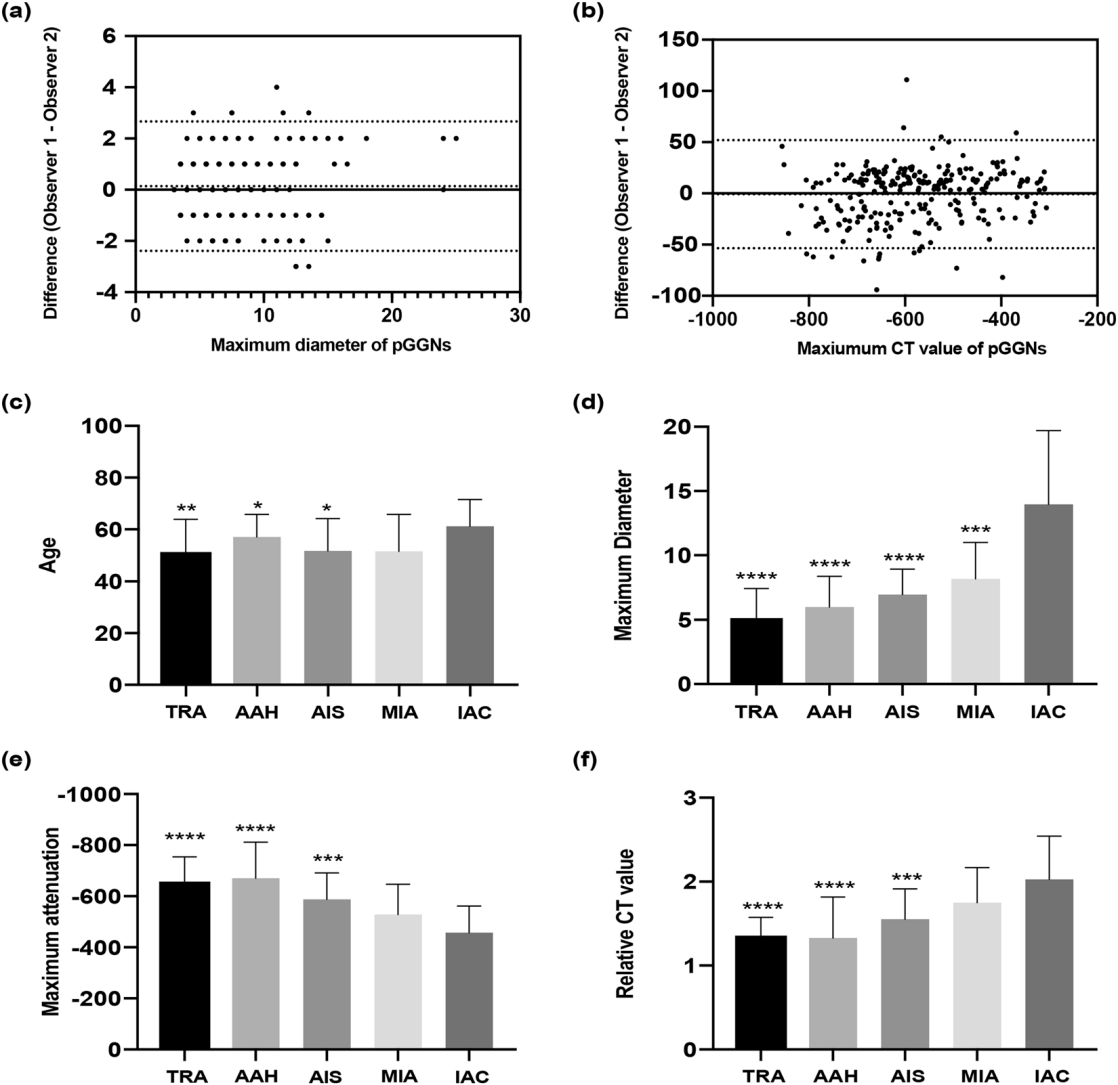
(**a**∼**b**) Bland-Altman plots showing Inter-observer agreement of measurements of the maximum diameter (**a**) and attenuation (**b**) of pGGNs. X axes show mean measurements and Y axes show differences between measurements of two observers. (**c**∼**f**) Distribution and multi-group comparisons (to take IAC as control group) of five pGGNs groups in parameters of age (**c**), maximum diameter (**d**), maximum attenuation (**e**), and relative attenuation (**f**). (Significant level marks: p < 0.05 *p < 0.01 **p < 0.001 ***p < 0.0001****).

Inter-observer agreement was excellent for maximum diameter of pGGNs (ICC range: 0.92–0.95) and maximum CT value (ICC range: 0.97–0.98). Bland-Altman plots with 95% limits of agreement are shown in Fig. 5a,b.

### Multiple comparisons

To take the IAC group as the control (Fig. 5c–f), significant differences were detected between the TRA/AAH/AIS and IAC groups in terms of age; additionally, highly significant differences were detected between the TRA/AAH/AIS/MIA groups and the IAC group regarding the maximum diameter. Maximum attenuation, relative attenuation, and difference attenuation were highly significantly different between the TRA/AAH/AIS and IAC groups. However, no significant differences were detected between the MIA and IAC groups in age, maximum attenuation, relative attenuation, or difference attenuation.

To take the TRA group as the control (Table 2), significant differences were detected between the TRA and IAC groups in age and between the TRA and AIS/MIA/IAC groups in maximum diameter, maximum attenuation, difference CT value, and relative CT value. No significant differences were detected between the TRA and AAH groups in any radiologic features.

**Table 2:** Multi-group comparison taking TRA as a control group.

	K-W statistic/Mean rank diff.	P
Age	13.47^a^	0.009^**^
TRAs vs. IACs	−56.49^b^	0.006^**^
Maximum diameter	90.77^a^	0.000^****^
TRAs vs. AISs	−59.74^b^	0.000^***^
TRAs vs. MIAs	−83.3^b^	0.000^****^
TRAs vs. IACs	−143.9^b^	0.000^****^
Maximum diameter	64.77^a^	0.000^****^
TRAs vs. AISs	−40.61^b^	0.031^*^
TRAs vs. MIAs	−74.35^b^	0.000^****^
TRAs vs. IACs	−114.2^b^	0.000^****^
Difference CT value	66.7^a^	0.000^****^
TRAs vs. AISs	−42.92^b^	0.019^*^
TRAs vs. MIAs	−76.79^b^	0.000^****^
TRAs vs. IACs	−114.5^b^	0.000^****^
Relative Value	67.55^a^	0.000^****^
TRAs vs. AISs	−42.71^b^	0.020^*^
TRAs vs. MIAs	−76.87^b^	0.000^****^
TRAs vs. IACs	−116^b^	0.000^****^

^a^Kruskal-Walls test value; ^b^Dunnett t-test value.

### Comparisons between two sample groups and ROC analysis

To take the TRA, AAH, and AIS groups as the non-progressive group (n = 123, males 40, 52 ± 12 years), the MIA and IAC groups as the progressive group (n = 132, males 40, 54 years, range, 16–80 years), highly significant differences were identified in maximum CT value, maximum diameter, relative CT value and difference CT value (Table 3). The mean or median values of the maximum diameter of the non-progressive and progressive groups were 6 mm and 8 mm, respectively, and the difference was significant, with an optimal cut-off value of 6.5 mm. The mean maximum CT values of the non-progressive and progressive groups were −646 HU and −515 HU, with an optimal cut-off value of −532 HU exhibiting 57.6% sensitivity and 86.2% specificity. Progressive pGGNs were more likely to be round or oval in shape, with clear margins, air bronchograms, vascular and pleural changes, creep growth, and bubble-like lucency than were non-progressive pGGNs.

**Table 3:** Radiologic characteristics of study population in non- and progressive groups.

Group	Non-progressive	Progressive	F	p
Cases	123	132	—	—
Age (years)	52 ± 12	54 (44–61)	—	0.273
Gender male [%]	40 [32.5]	40 [30.3]	0.145	0.073
Lobe location (LL, %)	47 [38.2]	44 [33.3]	3.123	0.537
Band Location (inner, %)	17 [13.8]	17 [12.9]	1.328	0.515
Maximum diameter (mm)	6 ± 2	8 (6–11)	—	0.000
Shape (round/oval, %)	26 [21.1]	62 [47]	18.798	0.000
Margin (clear, %)	63 [51.2]	98 [74.2]	16.181	0.000
Air bronchogram	5 [4.1]	26 [19.7]	14.569	0.000
Vessel change	17 [13.8]	89 [67.4]	77.527	0.000
Pleural change	4 [3.3]	23 [17.4]	13.508	0.000
Creep growing sign	11 [8.9]	52 [39.4]	31.738	0.000
Bubble-like lucency [%]	1 [0.8]	20 [15.2]	17.322	0.000
Maximum CT value	−646(−119–575)	−515 ± −119	—	0.000
Difference CT value	233(171–299)	367(268–446)	—	0.000
Relative CT value	1.36 (1.23–1.51)	1.72 (1.43–2.04)	—	0.000

**Figure 6:**
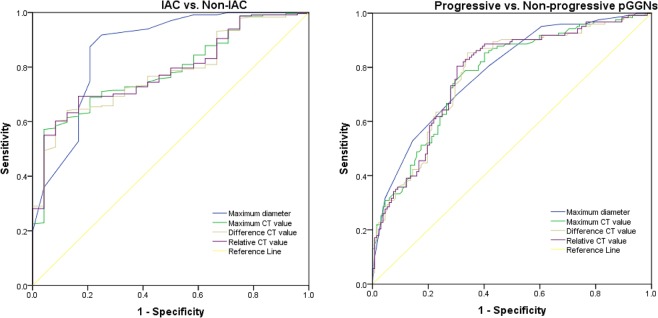
Graphs show ROC curves for maximum diameter and attenuation parameters for prediction of IAC (**a**) and progressive adenocarcinoma (**b**).

Receiver operating characteristic (ROC) analysis for identifying IACs among the pGGNs (Table 4) demonstrated the highest area under the curve (AUC) for the maximum diameter (0.865, 95% confidence interval, 0.776–0.955). The cut-off value of the maximum diameter was 11.5 mm (sensitivity, 75%; specificity, 91.8%). The cut-off value of the maximum CT attenuation was −574 HU (sensitivity, 95.8%; specificity, 57.1%). The relative CT value and difference CT value had similar diagnostic performance as the maximum CT attenuation (Fig. 6).

**Table 4:** ROC analysis of the maximum diameter and attenuation measurements on non-enhanced CT scans to discriminate IAC and progressive pGGNs.

Groups	Area	Std.Error	Asymplotic Sig.b	Cut-off Value			Asymptolic 95% Confidence Interval	
				Cut-off Value	Sensitivity (%)	Specificity (%)	Lower Bound	Upper Bound
**IAC& None-IAC**								
Maximum diameter	0.865	0.046	0.000	11.5	75	91.8	0.776	0.955
Maximum CT value	0.784	0.041	0.000	−574.5	95.8	57.1	0.703	0.864
Difference CT value	0.777	0.04	0.000	308.5	91.7	60.2	0.698	0.856
Relative CT value	0.784	0.04	0.000	1.54	91.7	60.2	0.706	0.862
**Progressive& None-progressive**								
Maximum diameter	0.783	0.028	0.000	6.5	69.7	30.1	0.728	0.838
Maximum CT value	0.722	0.029	0.000	−532	57.6	86.2	0.715	0.83
Difference CT value	0.774	0.029	0.000	323	65.9	85.4	0.716	0.831
Relative CT value	0.776	0.029	0.000	1.57	66.7	82.9	0.719	0.834

### Univariate and multivariate analyses

The characteristics of pGGNs were evaluated using univariate and multivariate analyses with logistic regression to assess their ability to predict progressive adenocarcinoma and IAC (Table 5). The following parameters were significantly associated with a high risk of progressive adenocarcinoma: maximum diameter, shape, margin, air bronchogram, vascular and pleural changes, creep growth, and maximum CT value. In addition to the parameters above, sex was a predictive parameter for IAC, although it was not a risk predictor for progressive pGGNs. The relative and difference CT values were not analysed in logistic regression because these parameters displayed no difference in diagnostic efficiency from that of maximum CT attenuation.

**Table 5:** Univariate and multivariate analysis of potential and significant predictors of progressive adenocarcinoma and IAC.

	Univariate^a^ (*p*)	Progressive & None-progressive			Univariate^a^ (*p*)	IAC & None-IAC		
		OR	95% CI	*p*-value		OR	95% CI	*p*-value
Age	0.613	1.03	0.97–1.10	0.31	0.002	1.00	0.97–1.03	0.85
Gender	0.703	0.22	0.05–0.93	0.04	0.015	1.53	0.65–3.61	0.34
Location	0.138	1.38	0.79–2.42	0.26	0.606	0.94	0.72–1.24	0.68
Distribution	0.357	0.73	0.26–2.02	0.55	0.290	1.12	0.64–1.98	0.69
Nodule size	0.000	1.63	1.24–2.14	0.00	0.000	1.32	1.13–1.53	0.00
Shape	0.000	0.59	0.10–3.58	0.56	0.001	4.24	1.44–12.44	0.01
Margin	0.004	0.86	0.27–2.80	0.80	0.635	0.19	0.09–0.41	0.00
Air bronchogram	0.001	4.10	0.71–23.49	0.11	0.000	1.54	0.33–7.15	0.58
Vessel change	0.000	1.06	0.35–3.24	0.92	0.000	9.45	3.94–22.68	0.00
Pleural change	0.001	6.12	1.30–28.85	0.02	0.001	4.96	0.93–26.29	0.06
Creep growing sign	0.016	0.97	0.21–4.47	0.97	0.023	0.31	0.11–0.83	0.02
Maximum CT value	0.000	1.01	1.01–1.02	0.00	0.000	1.01	1.01–1.02	0.00

CI = confidence interval; OR = odds ratio.

## Discussion

The results of our study demonstrated that middle and peripheral location, large maximum diameter and attenuation, bubble-like lucency, round or oval shape, clear margin, air bronchogram, vascular and pleural changes and creep growth were predictive of malignancy in pGGNs, while smaller diameter and maximum attenuation were associated with benignity. In our study, the prevalence of malignant pGGNs was 51.8%, and the majority of these cases were MIAs (43.4%), which is in accordance with the results of the study of Wu *et al*.^8^, who identified invasive lesions in 55.3% of sub-centimetre pGGNs. Henschke *et al*.^9^ reported a malignancy rate of 63% in partially solid nodules and 18% in pGGNs. The difference is plausible given that our study population included pGGNs that were subjected to surgical resection, which might have led to selection bias, and a large majority of persistent benign pGGNs were being follow-up and pathologically unconfirmed. A large number of studies have demonstrated the relationship between the size of pGGNs and pathological subtypes with a cut-off value of 10 mm for predicting malignancy^10–13^. In our study, the maximum diameter of progressive adenocarcinoma was significantly larger than that of TRA and preinvasive lesions and exhibited an increasing trend according to pathologic progression from AAH to IAC. The optimal cut-off values of the maximum diameter were 6.5 mm and 11.5 mm for differentiating non-progressive from progressive pGGNs and IAC from non-IAC, respectively. There was no difference in diameter between TRA nodules and adenomatous hyperplastic nodules.

Si *et al*.^14^ suggested the lack of significant difference in mean CT attenuation, lobe location and the presence of bubble-like lucency among 60 pathologically confirmed pGGNs. In our study, maximum CT attenuation, difference CT value, and relative CT value were good predictors of progressive adenocarcinoma. The reason for this discrepancy may be that the average CT value was measured in the study by Si *et al*., which may include small vessels within the nodule and fail to reflect areas of cysts and fibroplasia in pGGNs. Kim *et al*.^15^ indicated that any vessels penetrating or adjoining the nodule can substantially increase nodule attenuation and size; thus, we performed our measurements five times and took the crest value as the maximum CT attenuation of the nodule to avoid the effect of small vessels. Additionally, the relative and difference CT values were no more effective than the maximum CT attenuation in predicting the risk of pGGNs, indicating that different scanning machines do not affect the predictive ability of maximum CT attenuation. Several studies^16–18^ have reported that the size of the solid component is a better prognostic predictor than the size of the whole nodule, as the former reflects the invasive component. For pGGNs that lack visible solid components on TSCT, the maximum CT attenuation of the nodule, rather than solid components, may reflect the areas of dense tumour cells and the proliferation of fibroblasts inside the nodule, which may be relate to malignancy^19^. Koei *et al*.^20^ analysed a histogram of CT values of 43 GGNs and reported that −584 HU was the optimal cut-off value for differentiation between AAH and bronchioloalveolar carcinoma (BAC), which is lower than the optimal cut-off of maximum CT value in our study (−532 HU). The reason may be that for this discrepancy, Koei *et al*. used an automatic calculation method and considered the 75^th^ percentile CT value as the attenuation of GGNs. Therefore, not only the tumour size but also the maximum attenuation is associated with the malignancy of pGGNs, and thus, nodules with a maximum CT value > −532 HU, regardless of whether they have a large maximum diameter, should be followed up regularly.

The pathological basis of pGGNs is alveolar epithelial hyperplasia, an increase in the number of cells in the alveoli, thickening of the alveolar septum and fluid accumulation in the bronchiole terminals. Therefore, with the increase in the proportion of soft tissues in the pGGNs, pathological severity and CT attenuation are also increased. Because AAH is a localized, small, proliferative lesion caused by mild to moderate atypical hyperplasia of type II alveolar and/or Clara cells arranged on the alveolar wall or on the wall of respiratory bronchioles, there is more air and less cellular components in AAH, and thus, AAH has a lower CT value. However, TRA lesions mainly appear as inflammatory effusion in pathology, and soft tissue components in the lesions are relatively rare. Thus, in this study we failed to distinguish TRA nodules from adenomatous hyperplastic nodules.

Kuhlman *et al*.^21^ suggested that bubble-like areas due to small air-containing bronchi within lung nodules are characteristic enough to predict lepidic growth of adenocarcinomas. Yanagawa *et al*.^22^ showed that air bronchograms with disruption and/or irregular dilation were significantly more prominent in progressive adenocarcinoma than AIS. In our study, bubble-like lucency and air bronchograms were more prominent in progressive adenocarcinoma, which was in accordance with the results of previous studies. Histologically, this may be associated with the lepidic growth of adenocarcinoma on emphysematous lesions, the growth of adenocarcinoma along a pre-existing bullous emphysema, or a “check-valve” mechanism of small airway dilation with fibrous tissue^23^.

The appearance of vascular change, including small vessel convergence and dilation, were good predictors for progressive adenocarcinoma in our study, consistent with the reports of many studies^24–26^. In our study, creep growth was a newly identified feature in a majority of MIA and IAC cases. Histologically, these pGGNs are more likely to be located next to the adventitial membrane of larger vessels, with no gap between the nodule edge and the vessel adventitia; thus, on TSCT images, the boundary between the edge of the pGGN and the vessel adventitia is clear.

TRA GGNs, which spontaneously disappear during follow-up, are a manifestation of benign lesions, involving inflammation, haemorrhage, and infiltration with eosinophils^14^. In our study, maximum diameter, maximum attenuation and morphological characteristics could help distinguish TRA lesions from MIA and IAC, but we could not find a predictor to differentiate between TRA nodules and adenomatous hyperplastic nodules.

Our study has several limitations. First, the retrospective nature of this study might have induced selection bias. Second, we manually calculated the maximum diameter of pGGNs, which may lead to a lack of reproducibility by computer-aided volumetric software. Third, we failed to discuss whether some clinical parameters, such as smoking, BMI, and tumour markers, differed between the subtypes of pGGNs because baseline information was not available in our picture archiving and communication system. These limitations should be addressed in future research.

In conclusion, TSCT morphologic features and attenuation are helpful in differentiating among subtypes of pGGNs and may also be used to predict pathological invasiveness.
